# An In Vitro Study on the Efficacy of Green Synthesized Silver Nanoparticles on Surgical Site Infections and Healings

**DOI:** 10.3390/biomedicines14030634

**Published:** 2026-03-12

**Authors:** Gürkan Güneri, Merve Keskin

**Affiliations:** 1 Faculty of Medicine, General Surgery Department, Bilecik Şeyh Edebali University, Bilecik 11230, Türkiye; 2 Vocational School of Health Services, Bilecik Şeyh Edebali University, Bilecik 11230, Türkiye; merveozdemirkeskin@gmail.com

**Keywords:** surgical site infections, silver nanoparticles, antibiotic resistance, antimicrobial activity, wound healing

## Abstract

**Background/Objectives**: The healing rate of wounds resulting from postoperative abdominal surgery interventions increases as the inflammation and infections in the wound are reduced. However, antibiotic resistance among microorganisms increases the incidence of surgical site infections (SSIs). Therefore, the need for new products that exhibit antimicrobial and anti-inflammatory activities, in addition to antibiotics, is increasing. **Methods**: Silver nanoparticles (CO-AgNPs) were obtained using the green synthesis technique with *Cydonia oblonga* L. leaves, which constitute a significant amount of waste, and the effects of the obtained nanoparticles on in vitro wound healing were determined. **Results**: It was observed that CO-AgNPs inhibited myeloperoxidase and collagenase, enzymes that negatively affect wound healing. Furthermore, they exhibited good antimicrobial activity against *Pseudomonas aeruginosa*, *Escherichia coli*, *Bacillus subtilis*, and *Staphylococcus aureus*, which are common hospital pathogens. The CO-AgNPs could exhibit enhanced wound-healing properties by inhibiting microorganisms. **Conclusions**: It was clear that the development of new, environmentally friendly, and biocompatible products containing CO-AgNPs could be feasible, particularly for wound healing following infected abdominal surgery.

## 1. Introduction

Surgical site infections (SSIs) are defined as infections occurring at or near the surgical incision within 30 days after an operative procedure, or within 90 days in the presence of prosthetic material or implant placement [1]. SSIs are one of the most common complications of abdominal surgery. Despite advances in surgical techniques, pre- and post-operative care, and antimicrobial prophylaxis, this complication continues to escalate, particularly in abdominal surgeries requiring emergency surgery [2]. These infections significantly increase morbidity and mortality due to prolonged hospital stays, extended antimicrobial therapy, increased need for re-intervention, and management of serious postoperative complications, thus creating a significant clinical and economic burden [3,4]. Surgical site infections, beyond their harmful local effects such as impaired and delayed wound healing, frequently trigger systemic inflammatory responses that can progress to sepsis and organ failure, particularly in high-risk and vulnerable patient groups. Contamination, particularly from emergency gastrointestinal surgery, when combined with temporary immunosuppression, significantly increases the risk of postoperative infection and complications. Strong evidence from the literature shows that postoperative surgical site infections are independently associated with a significant increase in hospital stay, readmission rates, and mortality rates [5,6].

One of the most significant problems with SSI is antibiotic resistance. Antibiotic resistance has been identified by the WHO as one of the greatest threats to humanity and is predicted to be responsible for 10 million deaths by 2050 [7,8]. Antibiotic resistance renders surgical site infection treatment ineffective, increasing morbidity and mortality [9]. Specifically, biofilm-forming pathogens increase resistance to conventional treatments by limiting antibiotic penetration and lead to chronic infections in the surgical wound environment [10,11]. This means that the development of new antibiotics alone is insufficient to control infections; there is also a need for alternative agents to address antimicrobial resistance [12]. It has also been reported in the literature that reducing myeloperoxidase (MPO) and collagenase activity affects wound healing after complex surgical procedures. High MPO activity causes increasing intracellular oxidative stress and inhibits wound healing [13,14]. In addition, high collagenase activity degrades tissue structure and delays healing by preventing new tissue formation in chronic wounds [15]. As a result, developing new products with antimicrobial, antioxidant, and anti-inflammatory properties could help prevent and treat surgical site infections [16,17].

Silver nanoparticles (AgNPs) possess unique surface properties that enhance their biological activities [18]. AgNPs interact with microorganisms’ cell membranes, increasing membrane permeability and the cell’s osmotic pressure, leading to cell death [19,20]. AgNPs could be synthesized via chemical and biological (green) methods. However, chemical methods are harmful to the environment and human health due to toxic chemicals used in the process [21,22,23]. For this reason, green synthesis has been popular in recent years [22,23]. In this method, plants, plant waste, fungi, and algae are used to reduce ionic silver to metallic silver and stabilize AgNPs [24]. Room temperature and low energy consumption are used because of the hydroxyl and carbonyl groups present in biological materials. The metabolites act as both electron donors and stabilizers, which provides a controlled manner in the synthesis [25].

In this study, *Cydonia oblonga* L. leaves-based AgNPs were synthesized, characterized, and biological activities such as antioxidant, antimicrobial (on *E. coli*, *P. aeruginosa*, *S. aureus*, and *B. subtilis*), and enzyme inhibition properties on myeloperoxidase and collagenase were determined to find out the potential use of AgNPs in surgical wound healing.

## 2. Material and Methods

*Cydonia oblonga* L. plants were obtained commercially from Bilecik (Türkiye). A Milwaukee (USA) brand pH meter (Milwaukee Instruments, Inc., Rocky Mount, NC, USA) was used to adjust pH solutions. All chemicals were purchased from Sigma-Aldrich (Istanbul, Türkiye) in analytical grade.

### 2.1. Synthesis and Characterization of Cydonia oblonga L.-Based Silver Nanoparticles

*Cydonia oblonga* L.-based silver nanoparticles (CO-AgNPs) were obtained as described by Keskin [26]. The dried leaves of *Cydonia oblonga* L. were extracted with distilled water at a 1:10 (*w*/*v*) ratio, and the extract was mixed with a 0.005 M AgNO_3_ (in a ratio of 1:1 (*v*/*v*)) at room temperature and at a constant speed. The optimum pH was determined by preparing the extract with different pH solutions (pH 5.0, pH 7.0, and pH 9.0). The CO-AgNPs were synthesized separately in each solution, and their absorbance was measured using a UV-Vis spectrophotometer (Thermo Fisher, Waltham, MA, USA). The CO-AgNPs were centrifuged at 9000 rpm for 15 min, and the impurities were removed by washing the particles with distilled water. The CO-AgNPs were dried (75 °C, 12 h) [26]. To characterize CO-AgNPs, a UV-Vis spectrophotometer (for optical properties), Fourier Transform Infrared Spectroscopy (FT-IR, Perkin Elmer/SPECTRUM 100, Waltham, MA, USA for functional groups), and Scanning Electron Microscope (SEM, ZEISS/Supra 40 VP, Heidelberg, Germany, for the size) were used.

### 2.2. Antioxidant Activities of CO-AgNPs

DPPH· radical (2,2-diphenyl-1-picrylhydrazyl) scavenging activity was determined according to [27]. To determine radical-scavenging activity, the DPPH· extract in methanol was mixed with a sample solution prepared at different concentrations (100 to 500 µg/mL) in a 1:1 (*v*/*v*) ratio. The mixtures were incubated for 50 min, and the absorbance was recorded at 517 nm at the end of the period. The absorbance values were plotted and expressed against the ascorbic acid standard. The analyses were repeated in triplicate, and % inhibition values were calculated according to Formula (1) below.

% inhibition = (Acontrol − Asample)/Acontrol × 100(1)

Iron Reduction Capacity (FRAP) of CO-AgNPs was described by [28]. In this method, the reducing iron(III) ions in the Fe(III)-TPTZ complex could be determined by UV-vis spectroscopy. The freshly prepared FRAP agent, iron(III) chloride (FeCl_3_), buffer solution in 40 mM HCl, and 20 mM iron(III) chloride hexahydrate (FeCl_3_.6H_2_O) were mixed. The 3 mL of FRAP reagent and 0.1 mL of sample were mixed and incubated for 4 min at 37 °C. After incubation, the reducing absorbance of Fe(III) was recorded at 595 nm. The analysis was performed for different concentrations of sample solutions. Ascorbic acid was used as a standard. The analyses were repeated in triplicate, and % inhibition values were calculated according to Formula (1).

### 2.3. Collagenase Inhibition

To determine collagenase inhibition, the collagenase enzyme was dissolved in a 50 mM Tris-HCl (pH 7.5) buffer and incubated with CO leaves extract and CO-AgNPs in a buffer containing 10 mM CaCl_2_ and 400 mM NaCl [29]. Then, 0.8 mM N-(3-[2-Furyl]acryloyl)-Leu-Gly-Pro-Ala (FALGPA) was added to the mixture and incubated at 37 °C for 20 min. Absorbance changes were recorded at 340 nm, graphed, and percent inhibition values were calculated. Oleanolic acid was used as a standard inhibitor [29].

### 2.4. Myeloperoxidase (MPO) Inhibition

To determine myeloperoxidase inhibition by CO leaf extract and CO-AgNPs, pre-incubation of myeloperoxidase (2.5 nM) was performed in 50 mM phosphate buffer (pH 7.4) containing 0.5 mM H_2_O_2_. Subsequently, the CO leaves extract was removed, and CO-AgNPs were added separately to the mixture for 15 min. Afterward, 1 mM guaiacol (substrate) solution was added to the reaction cells at 37 °C, and the absorbance was recorded at 470 nm. Quercetin was used as a standard inhibitor, and the % inhibition values were calculated [30].

### 2.5. Determination of the Antimicrobial Activities of CO-AgNPs

The antimicrobial activity of CO-AgNPs was performed according to Cornican et al. [31]. *E. coli*, *B. subtilis*, *P. aeruginosa*, and *S. aureus* strains were individually cultured on Mueller–Hinton Agar (MHA) at 37 °C for 24 h. The overnight-cultured organisms were diluted in 0.9% *w*/*v* saline for a 0.5 McFarland and 100 μL of this culture was spread onto an agar plate. Afterward, sterile discs were placed on the agar, and 100 µL of the CO-AgNPs solution (20 mg/mL) was absorbed onto the discs. After incubation at 37 °C for 24 h, the inhibition zone diameter was measured in millimeters (mm). All experiments were repeated three times for each microorganism. Ampicillin was used as a positive control.

### 2.6. Statistical Analysis

All experiments were conducted in triplicate, and the results were presented as mean ± standard deviation (SD). Statistical significance was assessed at *p* ≤ 0.05, and mean differences were evaluated using Duncan’s multiple range test (DMRT).

## 3. Results and Discussion

**Figure 1:**
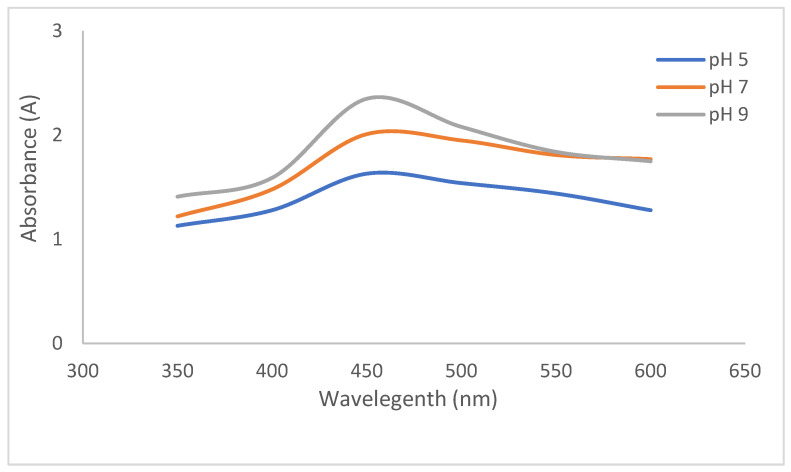
UV-Vis spectrum of CO-AgNPs and effect of pH.

The optical properties of the CO-AgNPs were determined using a UV-Vis spectrophotometer, and the maximum absorbance was found at nearly 450 nm (Figure 1). In general, AgNPs show maximum absorbance between 400 and 450 nm. The maximum absorbance value varies due to different reasons, such as plant material and synthesis conditions.

**Figure 2:**
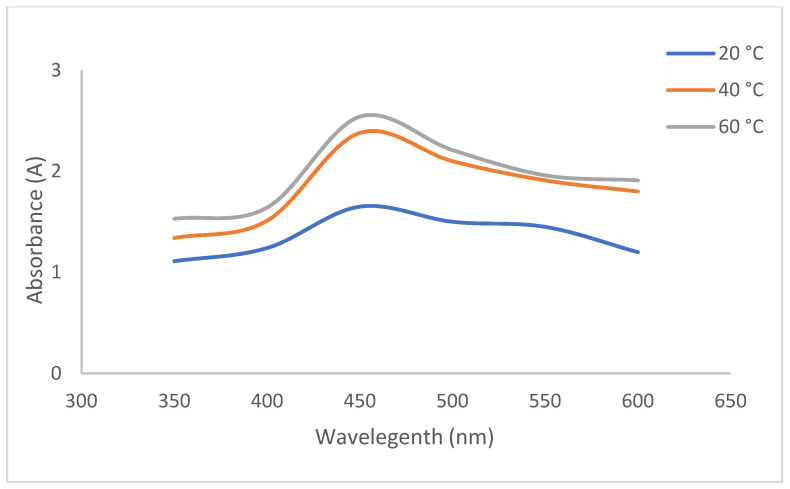
The effect of temperature on the synthesis of CO-AgNPs.

For example, in a study, *Glycyrrhiza glabra* Linn-based AgNPs exhibited maximum absorbance at 412 nm and 403 nm [32]. In another study, *Nigella sativa-based* AgNPs exhibited maximum absorbance at 450 nm [33]. Another parameter that affects the synthesis is pH. At different pH values, the functional groups in metabolites exhibit different ionization capacities, which affect electron donation. As a result, under basic conditions, the functional groups reach maximum ionization, thereby reducing Ag^+^ to Ag^0^ more effectively. In this study, absorbance was maximized at pH 9 (Figure 1), consistent with the literature [34,35,36]. Temperature is another factor that affects the synthesis of AgNPs. It was reported that at high temperatures, more homogeneous, smaller nanoparticles are obtained [37]. The optimum absorbance temperature was found to be 90 °C (Figure 2), but the synthesis was performed at room temperature due to metabolite degradation in plant material and to minimize energy use.

**Figure 3:**
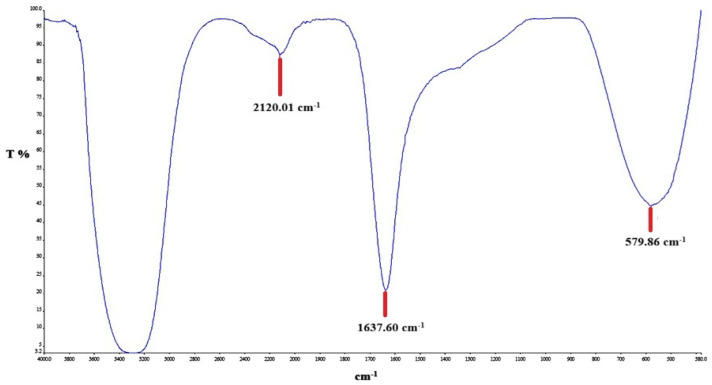
FTIR spectrum of the CO leaves extract.

**Figure 4:**
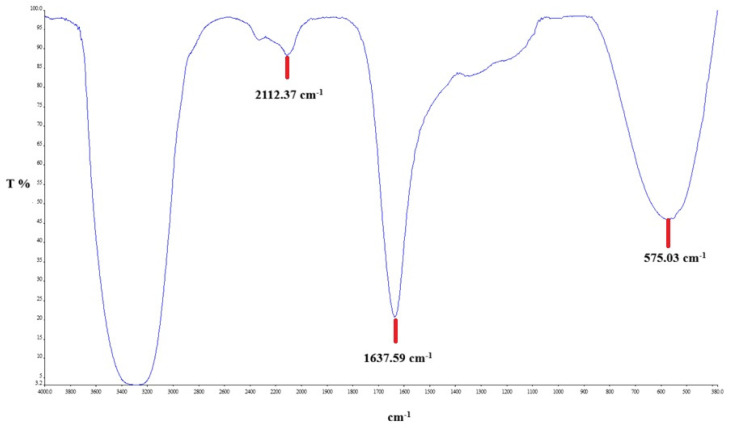
FTIR spectrum of CO-AgNPs supernatant.

FTIR analysis was performed to identify the functional groups present in the production of silver nanoparticles. Figure 3 and Figure 4, and Table 1 show the spectra of the CO leaves extract and the CO-AgNPs supernatant. Accordingly, the strong and broad band at 3200–3400 cm^−1^ corresponds to –OH vibrations, indicating the presence of phenolic compounds, alcohols, and proteins. The bands located at 1600–1650 cm^−1^ indicate the presence of C=O (carbonyl). The peaks located at 1000–1200 cm^−1^ indicate C-O, C-N, or C-O-C vibrations. These peaks are associated with the presence of polysaccharides, flavonoids, and other plant metabolites. When examining Figure 4, shifts in these peaks are observed. This situation serves as evidence that the green synthesis has been successfully carried out.

**Figure 5:**
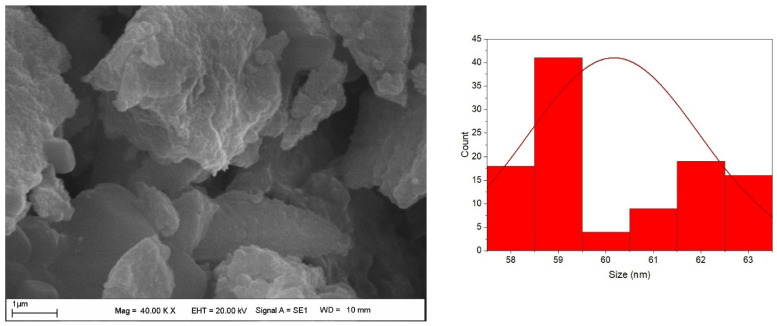
SEM image and histogram of CO-AgNPs.

**Figure 6:**
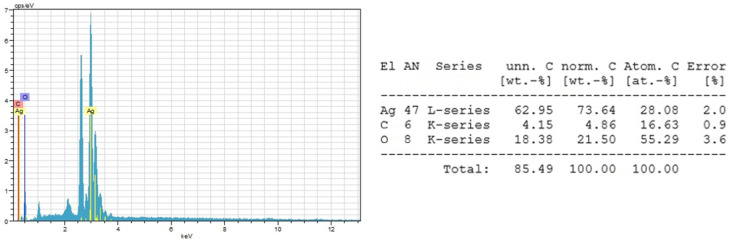
EDX and metallic profile of CO-AgNPs.

The sizes of CO-AgNPs were found between 58 nm and 63 nm (mean value 60 ± 2 nm) (Figure 5). The shape of the nanoparticles was generally spherical. The shape and size of AgNPs also vary with synthesis conditions and source. In a study, Centella asiatica-based silver nanoparticles ranged from 10 to 50 nm [38]. When the study was examined, it was found that the synthesis conditions (AgNO_3_ amount) affect the size of AgNPs. In this regard, it was reported that the size of AgNPs varies with plant material and synthesis conditions such as pH, extract, and AgNO_3_ concentrations, temperature, etc. [39,40,41]. The chemical composition of silver nanoparticles could be determined by energy-dispersive X-ray spectroscopy (EDX). The release of excess energy by the atoms in the sample due to an electron beam creates peaks in the spectrum. Individual elements may have multiple peaks, and peaks of different elements may overlap to some extent [42]. The sharp peak at around 3 keV is due to silver. The EDX profile of CO-AgNPs is presented in Figure 6. The sharp peak around 3 keV indicates that the synthesis of CO-AgNPs was successful.

**Figure 7:**
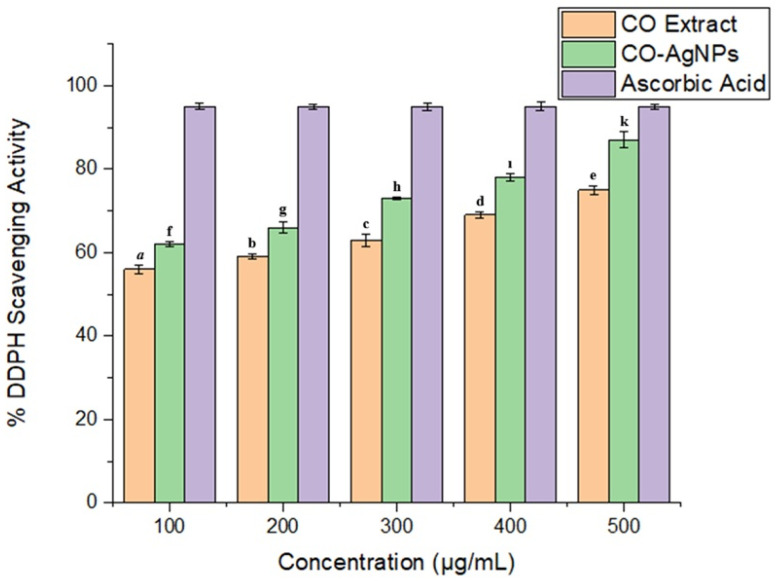
DPPH· scavenging activity of CO leaves extract and CO-AgNPs. Data were presented as means ± SD from three replicates. Different letters indicate a significant difference, while the same letter denotes no significant difference according to Duncan’s multiple range test at *p* ≤ 0.05.

**Figure 8:**
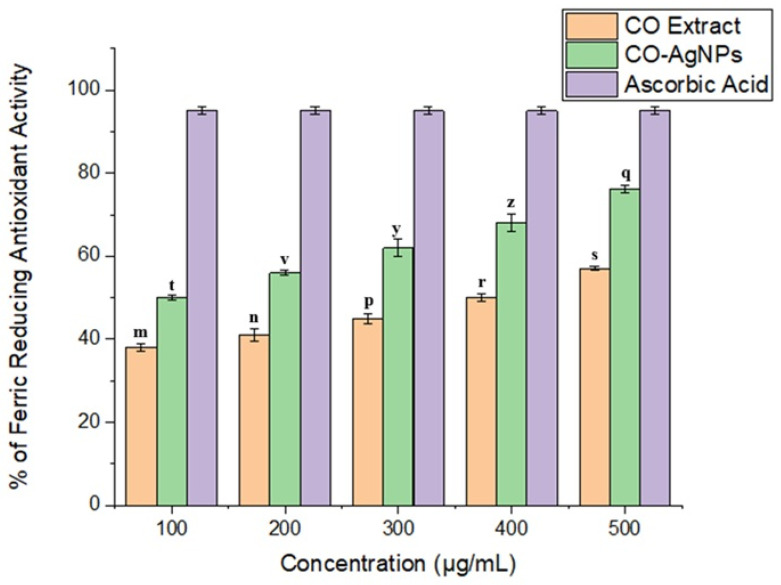
Ferric reducing activity of CO leaves extract and CO-AgNPs. Data were presented as means ± SD from three replicates. Different letters indicate a significant difference, while the same letter denotes no significant difference according to Duncan’s multiple range test at *p* ≤ 0.05.

The antioxidant capacity of CO-AgNPs was determined according to the DPPH· radical scavenging activity and FRAP iron reduction capacity. The results were 87 ± 2% and 76 ± 0.9% for 500 µg/mL CO-AgNPs, respectively (Figure 7 and Figure 8). It was clear that CO-AgNPs exhibited good antioxidant activity, and the antioxidant capacity increased with CO-AgNP concentration. It could be related that CO-AgNPs could affect reactive oxygen species (ROS), which are implicated in inflammation in the human body. Due to the proton-donation capacity of CO-AgNPs, they could act as free radical scavengers. In the literature, AgNPs have been reported to exhibit good antioxidant activity [43,44,45].

**Figure 9:**
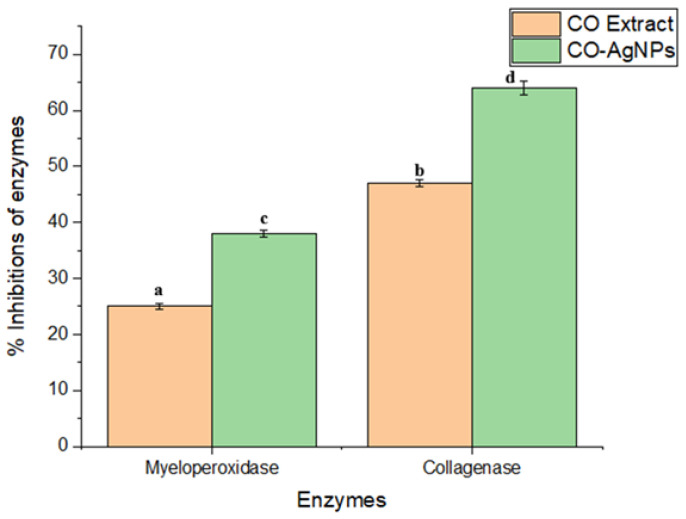
Inhibition effects of CO leaves extract and CO-AgNPs on myeloperoxidase and collagenase. Data were presented as means ± SD from three replicates. Different letters indicate a significant difference, while the same letter denotes no significant difference according to Duncan’s multiple range test at *p* ≤ 0.05.

The controlled inhibition of MPO and collagenase has a positive effect on wound healing. In this study, the inhibition effects of MPO and collagenase by CO-AgNPs were found to be 38 ± 0.8% and 64 ± 1.2%, respectively (Figure 9). The oleanolic acid inhibited collagenase 25.2 ± 0.9%, and the quercetin inhibited myeloperoxidase 48.6 ± 0.7%. It was clear that CO-AgNPs were more effective in inhibiting the enzymes. In a study, AgNPs were reported to inhibit MPO by 60% [46]. In another study by [47], the inhibition properties of myeloperoxidase and collagenase were determined to be 63% and 37%, respectively. In the literature, it was reported that AgNPs inhibit enzymes involved in wound-healing processes [48,49].

**Figure 10:**
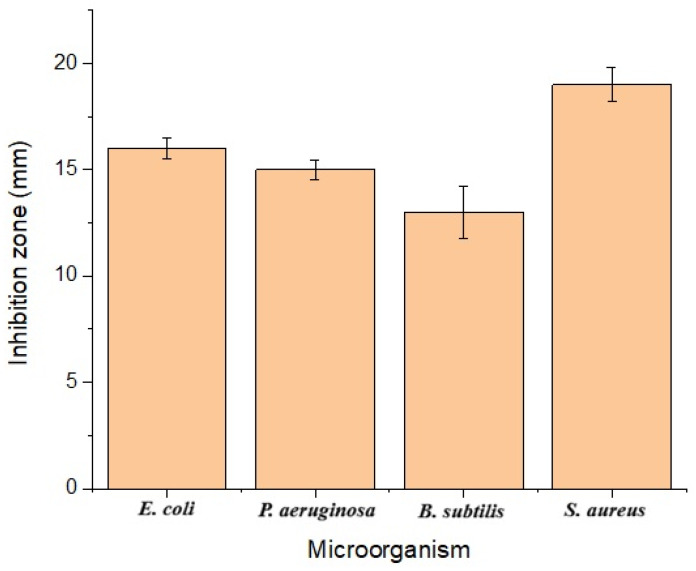
Antimicrobial activities of CO-AgNPs.

The antimicrobial activities of the synthesized nanoparticles against *E. coli*, *P. aeruginosa*, *B. subtilis*, *S. aureus*, and Ampicillin were found to be 16 ± 0.5 mm, 15 ± 0.5 mm, 13 ± 1.2 mm, 19 ± 0.8 mm, and 10 ± 0.5 mm, respectively (Figure 10). The minimal inhibitory concentration (MIC) was found to be 150 µg/mL for each microorganism. *E. coli*, *B. subtilis*, *P. aeruginosa*, and *S. aureus* are microorganisms commonly encountered in hospitals and are the most frequent cause of infection following surgical operations [50]. Therefore, inhibiting their growth is crucial as they can cause infection during wound healing. A study by [17] determined the effect of *Phoma glomerata*-based silver nanoparticles on *E. coli*, *P. aeruginosa*, and *S. aureus*, and found strong antimicrobial activity. In a study reported by [51], the effects of green-synthesized silver nanoparticles on *E. coli* and *B. subtilis* were determined. It was observed that the nanoparticles exhibited strong antimicrobial activity. Ref. [52] reported that mushroom-based silver nanoparticles exhibited antibacterial effects on *E. coli* and *B. subtilis*. The literature reports that silver nanoparticles obtained via green synthesis techniques exhibit high antimicrobial activity against various microorganisms [53,54,55]. Different studies on the green synthesis of AgNPs were compared with the literature in Table 2.

## 4. Conclusions

Preventing inflammation and infection during the healing of surgical wounds is crucial. However, the resistance that microorganisms have developed to antibiotics adversely affects this process. Therefore, there is a need to develop new products with high antimicrobial and anti-inflammatory effects. Silver nanoparticles exhibit antimicrobial and anti-inflammatory effects across a broad spectrum due to their unique properties. The use of silver nanoparticles synthesized in an environmentally friendly, biocompatible manner to promote wound healing after surgical operations will contribute to the healing process by preventing the development of antibiotic resistance. In this study, silver nanoparticles based on *Cydonia oblonga* L. were synthesized and characterized. The characterized silver nanoparticles exhibited antioxidant and antimicrobial activities, as well as inhibitory effects on myeloperoxidase and collagenase, enzymes that play important roles in wound healing. This in vitro preliminary study revealed that the synthesized nanoparticles exhibited excellent antioxidant and antimicrobial activity, whereas the enzymes were inhibited by more than 50%. The data obtained in this preliminary in vitro study indicate that the synthesized nanoparticles have the potential to be used to heal wounds after abdominal surgical procedures. However, additional analyses should be performed to determine the relationships among antioxidant, enzyme inhibition, and antimicrobial properties, and toxicity and biocompatibility should be tested in further studies. It also indicates that new products (e.g., wound dressings and cleaning solutions) incorporating these inexpensive and environmentally friendly nanoparticles can be designed.

## Figures and Tables

**Table 1:** FTIR peaks of the CO extract and supernatant.

Sample	I (cm^−1^)	II (cm^−1^)	III (cm^−1^)
CO Extract	2120.01	1637.60	579.86
CO-AgNPs supernatant	2113.37	1637.59	575.03

**Table 2:** Comparison of Co-AgNPs with other AgNPs that were synthesized via different sources.

Plant Source	Absorbance (nm)	Size (nm)	Application	Reference
*Glycyrrhiza glabra* Linn	412 and 403	69.7 to 419.1	Wound healing properties	[32]
*Nigella sativa*	450	69	Antibacterial activity and wound healing	[33]
*Mirabilis jalapa*	431–446	-	Antimicrobial activity	[56]
*Aloe vera*	420	255.5	Antibacterial activity and wound healing	[16]
*Centella asiatica*	310	10–50	Antibacterial activity and wound healing	[38]
*Verbascum splendidum*	402	40	Antibacterial activity and wound healing	[57]
*Cassia sericea*	237	50.4	Antibacterial activity and wound healing	[58]
This study	450	58 to 63	Antimicrobial activity and in vitro wound healing properties	-

## Data Availability

Data will be available when the request.
